# Mechanisms and Therapeutic Potential of Human Cardiomyocyte Proliferation

**DOI:** 10.3390/jcdd13020074

**Published:** 2026-02-02

**Authors:** Richard D. McLane, Abhay Cheruku, Ashley B. Williams, Ravi Karra

**Affiliations:** 1 Division of Cardiology, Department of Medicine, Duke University School of Medicine, Durham, NC 27710, USA; richard.mclane@duke.edu (R.D.M.); abhay.cheruku@duke.edu (A.C.); ashley.williams080@duke.edu (A.B.W.); 2 Department of Pathology, Duke University School of Medicine, Durham, NC 27710, USA

**Keywords:** cardiomyocyte proliferation, regeneration, heart failure

## Abstract

The limited capacity for cardiomyocyte proliferation in the adult human heart restricts its ability to recover from injury. Building on discoveries in regenerative model systems, such as zebrafish and neonatal mice, reactivation of a latent potential for cardiomyocyte proliferation is a strategy to promote therapeutic heart regeneration. Although cardiomyocyte proliferation remains modest even with the most effective mitogenic stimuli identified to date, evidence for a potential functional benefit in pre-clinical model systems has led to the initiation of several early-phase clinical programs. Here, we review insights from model organisms that inform the potential efficacy and limitations of therapeutic cardiomyocyte proliferation, systems to study human cardiomyocyte proliferation, and the natural history of cardiomyocyte proliferation in the human heart. We also examine the translational trajectory of selected discoveries, including therapeutic delivery modalities, and attendant safety concerns. Finally, we discuss critical challenges that will need to be addressed to enable successful clinical translation.

## 1. Introduction

Heart failure remains a growing public health challenge in the United States. Approximately six million Americans are affected, and this number is expected to rise as the population ages [1]. The economic burden is similarly substantial, exceeding USD 30 billion annually and projected to surpass USD 70 billion by 2030 [2]. Current pharmacologic and device-based therapies alleviate symptoms and prolong survival, yet they are not curative. With optimized guideline-directed therapy, patients with heart failure continue to face significant risk for recurrent hospitalization and death, underscoring the limitations of existing treatment paradigms. Even for the ~20% of patients who show improved cardiac function with medical therapy, these gains reflect clinical remission rather than true myocardial recovery [3,4]. Collectively, these clinical realities highlight a need for curative therapeutic approaches capable of rebuilding or replacing cardiac muscle.

A key barrier to myocardial repair is the adult human heart’s inability to meaningfully replace lost cardiomyocytes (CMs). For decades, this limitation was attributed to the prevailing view that CMs are terminally differentiated and incapable of proliferation. Early observations in adult newts in the 1970s suggested that CM proliferation was a mechanism for heart regeneration [5,6]. However, seminal work demonstrating that zebrafish hearts can regenerate after injury transformed the field [7]. Because zebrafish are genetically tractable, elegant lineage-tracing experiments definitively revealed that spared CMs near the injury site re-enter the cell cycle and divide to replace lost myocardium, rather than a resident stem cell population [8,9]. In 2011, neonatal mice were similarly shown to regenerate their myocardium after injury through CM proliferation, demonstrating that not only are mechanisms for regeneration conserved across species, but also that reawakening the capacity for CM proliferation in the adult heart could lead to therapeutic heart regeneration [10].

These discoveries, along with extensive work across diverse model systems, have reshaped long-held assumptions about CM plasticity and established the conceptual foundation for therapeutic heart regeneration. Since then, advances in developmental biology, tissue engineering, and gene therapy have propelled efforts to translate CM proliferative mechanisms into first-in-human clinical studies targeting myocardial repair. In this review, we highlight experimental systems that enable the study of CM renewal, examine evidence for CM proliferation in the human heart, discuss strategies to facilitate therapeutic heart regeneration, and highlight barriers that impede their translation to humans.

## 2. Models of Heart Regeneration

CM cell cycle reentry is the major mechanism that drives CM regeneration after injury in zebrafish, axolotls, and neonatal mammals. Consequently, even the minimal capacity for adult human CM proliferation has inspired the possibility that adult human CMs can be coaxed into cell cycle reentry for therapeutic CM proliferation. Here, we will briefly review different models of heart regeneration, emphasizing potential human model systems.

### 2.1. Non-Human Models

#### 2.1.1. Zebrafish

Zebrafish are the classical model for adult tissue regeneration and are capable of tissue growth throughout life. Adult zebrafish regenerate their hearts following surgical amputation of the ventricular apex, genetic ablation of up to 60% of CMs, and cryoinjury [7,11,12,13]. Shortly after injury, immune cells, nascent vasculature, and epicardial cells infiltrate the wound and establish an environment that instructs CMs to proliferate and reconstitute lost myocardium [14]. This microenvironment is critical: interference with supporting cell types diminishes CM proliferation and leads to defects in regeneration. Several CM mitogens that are sufficient to drive CM proliferation during zebrafish heart regeneration are Vegfaa from endothelial cells and Nrg1 from epicardial cells and regulatory T-cells [15,16,17]. In addition to cell–cell interactions that drive heart regeneration, zebrafish have been instrumental in understanding the cell-autonomous changes in CMs that enable cell-cycle reentry. CMs “de-differentiate” by disassembling their sarcomeres and re-expressing developmental factors such as *gata4* and *tbx5a* [8,18]. Genome-wide profiling approaches have further revealed that CMs undergo global changes in gene expression, chromatin accessibility, metabolism, and protein biosynthesis during regeneration [19,20,21,22]. Notably, adult zebrafish CMs are primarily mononuclear and diploid, and repopulating the zebrafish heart with polyploid CMs reduces the capacity for heart regeneration [23]. Thus, the predominance of polyploid CMs in the adult human heart is likely to be a major obstacle for human heart regeneration.

#### 2.1.2. Mice

Adult mouse CMs, like adult human CMs, exhibit minimal proliferation following injury, whereas neonatal mouse CMs retain the ability to re-enter the cell-cycle and regenerate myocardium. Ablation of ~60% of CMs in embryonic day 9.5 mice results in a roughly 20% increase in Ki67^+^ CMs [24]. Remarkably, adult hearts subjected to developmental CM ablation are indistinguishable from unablated controls with respect to morphology and function, indicating effective regeneration. In groundbreaking work, Porrello et al. showed that neonatal mice at 1–2 days of age are capable of regeneration following resection of the left ventricular apex or ligation of the left coronary artery [10,25]. Like zebrafish, regeneration in neonatal mice occurs by proliferation of CMs and depends on supporting cell types, including endothelial cells and macrophages [26,27,28].

Neonatal mice lose cardiac regenerative capacity within the first week of life, coincident with terminal CM binucleation [10]. This transient regenerative window has sparked investigations into mechanisms underlying the loss of CM proliferative capacity. Since the initial description of neonatal heart regeneration in mice, the number of interventions to promote cycling of adult mouse CMs to restore function after injury has rapidly expanded. A comprehensive overview of these approaches was recently reviewed by Koopmans et al. [29]. Broadly, strategies for promoting CM regeneration include (1) re-expression of developmental mitogens; (2) forced expression of canonical cell cycle proteins; (3) shifting CM composition towards a mononuclear, diploid state; (4) shifting CM metabolism away from fatty acid oxidation; and (5) epigenetic reprogramming of CMs toward a more immature state. An important lesson learned from the work on mice is that CM proliferation remains extraordinarily inefficient, with even forced overexpression of oncogenes more likely to result in endoreplication rather than true proliferation [30]. Moreover, CM differentiation and proliferation are so intricately intertwined that unconstrained, widespread CM cycling is potentially toxic [31].

#### 2.1.3. Pigs

Like mice, neonatal piglets are capable of heart regeneration. Piglets less than two days of age subjected to experimental myocardial infarction are able to fully recover their function without evidence of scarring [32,33]. Immunohistochemical analyses demonstrate increased DNA synthesis and expression of cytokinesis markers in CMs after injury. Remarkably, this ability for regeneration is lost by three days of age, after which cardiac injury results in fibrosis, resembling the response of adult human hearts. Because the porcine heart closely approximates the human heart in size, anatomy, and physiology, adult pigs have been widely used to assess the translational potential and safety of regenerative therapeutics identified in mice. Overexpression of the cell-cycle gene *Ccna2*, knockdown of the Hippo pathway gene *Sav*, and local injection of the extracellular matrix protein Agrin each promote recovery of the adult porcine heart, with evidence for CM cell cycle reentry and reduced fibrosis [34,35,36]. The comparable heart rate and electrophysiologic properties of the porcine heart have been critical to understanding potential toxicities of regenerative therapeutics. For example, injection of *miR-199a* into the infarcted pig heart initially improves cardiac function but ultimately leads to sudden cardiac death, highlighting potential arrhythmogenic risks associated with sustained CM proliferation [37].

### 2.2. Human Models

**Figure 1:**
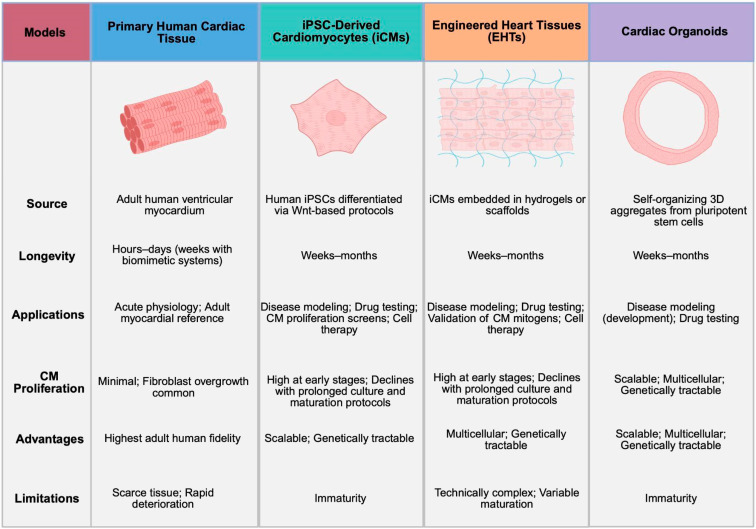
Comparison of various human model systems to study CM proliferation. Created in BioRender. McLane, R. (2026) https://BioRender.com/megafdt (accessed on 28 January 2026).

While non-human models have been invaluable to identifying mechanisms that govern CM proliferation, species-specific differences in CM biology and physiology raise the possibility that not all mechanisms are precisely conserved in human CMs. For example, human CMs tend to be ~70% mononuclear but with polyploid DNA content compared to zebrafish CMs, which are mononuclear and diploid, and mouse CMs, which are largely binucleate with variable ploidy [38]. Because polyploidy of CMs greatly influences the potential for true CM proliferation, CM mitogens identified in zebrafish and neonatal mice are unlikely to be as potent in the mature human heart [23,27,39]. The epigenetic programming between human CMs and mouse CMs differs. For example, *MYH7* is the predominant myosin isoform in adult human CMs, whereas *MYH6* dominates in fetal human CMs; the opposite is true in mice. This difference in isoform expression is probably mediated by differential utilization of enhancers in human and murine CMs during development and adulthood [40]. Similarly, different cocktails of transcription factors are needed to reprogram cardiac fibroblasts into CMs when using mouse or human cells [41]. Finally, murine and human CMs differ in their pharmacology, with unique complements of ion channels and G-protein coupled receptors [42]. Consequently, transcriptional programs or signaling pathways sufficient to induce cell-cycle activity in one species or developmental context may not be conserved in adult human CMs or may instead lead to qualitatively different outcomes, such as polyploidization. Accordingly, robust human models are needed to complement insights gained from non-human model systems to assess translational potential (Figure 1).

#### 2.2.1. Primary Human Cardiac Tissue

Culture of primary human cardiac cells has traditionally been limited by access to tissues. However, primary human ventricular CMs are now commercially available from various tissue banks. In general, primary human ventricular CMs are used for acute studies, as they lose their sarcomeric structure rapidly in culture. To overcome these challenges, various methods for immortalizing primary human ventricular CMs have been developed. In the AC16 cell line, primary human CMs were fused with SV40-transformed fibroblasts for immortalization [43]. Under basal conditions, these cells are proliferative, limiting their use for mechanisms of CM proliferation.

To model the cellular diversity of the human heart, methods for culturing cardiac slices have evolved over the past eight decades [44]. Early passive culture methods led to rapid tissue deterioration over hours to days, including loss of CM sarcomeric structure and T-tubules, as well as the expansion of resident cardiac fibroblasts [45]. Adding elements of in vivo physiology, such as pacing or mechanical loading of cardiac slices, can prolong culture times to ~5–6 days without sarcomeric deterioration, while combining both in a biomimetic system can extend culture times to ~4 months [45,46,47]. Ongoing refinements to media composition, like improved oxygenation and hormonal supplementation, may further enhance the longevity of cardiac tissue [44].

#### 2.2.2. iPSC-Derived Cardiomyocytes (iCMs)

The development of protocols to efficiently differentiate human induced pluripotent stem cells into relatively pure CM-like cells for extended periods has addressed a major need in studying human cardiac biology [48,49,50,51]. iCMs are widely used for diverse applications, including disease modeling, drug toxicity testing, understanding mechanisms of CM development, and as a source of therapeutic cardiac cellular replacement [52,53].

To study CM proliferation, iCMs have mostly been used to validate findings from other model systems in a human context and, more recently, as platforms to screen for proliferative compounds. Screening approaches typically quantify iCM nuclei or assess for cell-cycle activity using EdU incorporation, Ki67 immunostaining, or transgenic expression of cell-cycle reporters such as the FUCCI system [54,55,56,57,58,59,60]. Interestingly, these screens have largely converged on pathways previously implicated in CM proliferation such as the Hippo, mTOR, p38, and AMPK axes. Among the more de novo “hits,” inhibition of the Dyrk1a kinase or the fatty acid transporter Cpt1b promotes CM cycling in iCMs and has orthogonal effects in adult mice [61,62]. In support of human-specific mechanisms, a recent large screen of human miRNAs identified miRs in the primate-specific C19MC cluster as regulators of iCM proliferation [55]. However, several limitations complicate the use of iCMs for drug screening. First, as with other model systems, distinguishing true proliferation from endomitosis remains a challenge. Second, iCMs can have variable rates of cycling depending on differentiation conditions such as density and the parent cell line used [63]. Finally, and most importantly, iCMs more closely resemble fetal CMs: they are small and round, exhibit immature Ca^2+^ handling and electrophysiology, and have poorly organized sarcomeres [64,65]. As such, potential mitogens may have stronger effects on iCMs compared to adult human ventricular CMs. As better protocols are developed to mature iCMs in 2D and 3D systems, to identify true proliferation, and to make primary human tissue systems more robust and available, the identification of mitogens that specifically drive *human* CM proliferation is feasible.

#### 2.2.3. Engineered Heart Tissues (EHTs)

Human engineered heart tissues are generated when iCMs, usually in a hydrogel, are embedded within a three-dimensional scaffold or matrix to form tissue-like strips or patches. Methods for generating EHTs have recently been reviewed [66,67]. EHT scaffolds can be patterned and loaded onto elastomeric posts, resulting in CMs that are anisotropically aligned, like in native myocardium. Importantly, EHTs can be designed to incorporate endothelial cells, fibroblasts, neurons, macrophages, and other cell types, thus able to model aspects of intercellular cardiac interactions that 2D cultures of pure iCMs cannot. Compared to 2D cultures, EHTs can be matured with biomimetic cues like mechanical loading, pacing, and growth factors to harbor CMs that metabolically and structurally resemble in situ adult CMs. EHTs have been used for drug screening and disease modeling, in some cases capable of disease-relevant phenotypes that 2D systems do not [68,69].

The exact rate of CM proliferation in EHTs varies depending on the method used to generate them. Generally, less mature EHTs have higher basal rates of CM proliferation and even functionally recover after cryoinjury, with an associated increase in CM proliferation [70]. By contrast, more mature EHTs have less CM proliferation [71]. For example, metabolic maturation induced by the addition of palmitic acid, attenuates the rate of CM proliferation in EHTs [72]. Because of the complexity and scale of CMs needed to make EHTs, EHTs have largely been used to validate CM mitogenic cues identified in other systems like chemical inhibition of Wnt signaling, YAP activation, increasing endothelial content, or applying mechanical load [72,73,74,75]. Interestingly, the work on 2D systems and EHTs is not always concordant. For example, of 105 compounds found to increase EdU incorporation in 2D iCMs, only 83 had similar effects in 3D EHTs [76]. Importantly, this work went on to demonstrate that CM proliferation and loss of contractile function are separable by selectively activating the mevalonate pathway, findings that were replicated in mice and that demonstrate the importance of maturation in CM models.

#### 2.2.4. Cardiac Organoids

While there are varying definitions for cardiac organoids, here we consider cardiac organoids to be three-dimensional, self-organizing models of cardiac tissue generated from pluripotent stem cells [77]. Over the past several years, multiple protocols for generating cardiac organoids have been developed, ranging from forced aggregation of pluripotent cells into 3D embryoid body-like structures using patterned plates, using ultra-low attachment plates, or by embedding iPSCs in Matrigel followed by Wnt modulation [78,79,80]. Iterations that alter the timing and sequence of Wnt modulation, along with the incorporation of additional growth factors, have resulted in organoids with varying amounts of cellular diversity and structural complexity [79,81,82,83]. Additionally, organoids can be fused into complex structures, with these assembloids reminiscent of chambers [81]. Because their self-organization is largely based on developmental cues, cardiac organoids have primarily been used to study heart development and congenital heart disease. Compared to EHTs, CMs in cardiac organoids are less mature, but organoids generally do not require specialized platforms and can therefore be produced in a high-throughput fashion. CM proliferation rates are early in organoid formation and diminish with extended culture [79,82].

#### 2.2.5. Current Controversies and Limitations

While the number of approaches to coax adult mammalian CMs to re-enter the cell cycle has exploded over the past 10 years, very few of these interventions have been reproduced by multiple groups [29]. A major issue in the field is a lack of consensus on how to define and measure CM proliferation [30,84]. Marker-based approaches—such as those relying on EdU incorporation or the presence of Ki67, phospho-histone H3, or Aurora B kinase—are inherently limited because cycling adult mammalian CMs are much more likely to result in polyploid CMs rather than multiple new CMs. Even with these markers, the rarity of CM cycling creates additional challenges. Assigning a cycling nucleus to a CM is very difficult without using CM nuclear markers, for which the ideal CM nuclear marker remains controversial [84,85]. Additionally, analysis of a small number of sections or a small tissue area can lead to unstable estimates of CM cycling. Application of deep learning-based algorithms to assess large numbers of CMs may address some of these limitations [86,87]. To assess true cardiomyogenesis, several approaches have been proposed. One method is to count and assess DNA content in dissociated CMs, but the sensitivity of this approach for true proliferation is unclear [39,84]. Alternatively, lineage tracing of CMs to assess clonal expansion using rare labeling, multicolor lineage tracing, mosaic analysis of double markers or with combinatorial Cre/Dre-based systems is generally complex, with an uncertain sensitivity to assess rare events [88,89,90,91,92]. These genetic approaches, while theoretically able to identify new CMs, have few established methods for quantitatively assessing clonality [93]. Moreover, none of these genetic approaches have been widely used in human CM systems. Both technical innovations and consensus standards for measuring CM proliferation are very much needed.

## 3. Cardiomyocyte Proliferation in the Human Heart

### 3.1. Developmental Cardiomyocyte Proliferation

**Figure 2:**
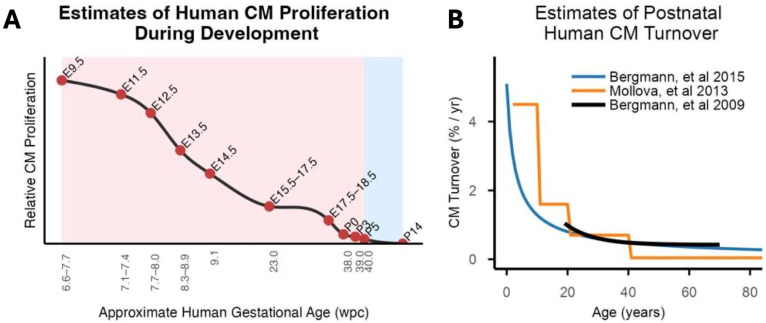
Estimated rates of human CM proliferation across the lifespan. (**A**). Relative rates of human CM proliferation at various developmental stages were extrapolated from data at matched stages in mice (points). Weeks post-conception (wpc) during human development are shown on the x-axis. Mapping between mouse and human developmental stages is based on the work of Krishnan, et al. [103]. Human prenatal stages are marked in pink, and postnatal stages are marked in blue. (**B**). Estimated rates of postnatal human CM proliferation based on data provided in references [104,105,106].

During early development, the heart undergoes rapid volumetric expansion, increasing from 2.7 × 10^−6^ µL at ~3 weeks post-conception (wpc) to ~26.6 mL by 30 wpc [94,95]. Because access to normal human embryonic heart tissue is difficult, insights into early cardiac growth dynamics have been inferred from morphologically matched mouse and chick models with support from a limited number of human embryonic and fetal samples (Figure 2A).

Developmental expansion of the CM pool proceeds through two principal modes of growth. The first involves the differentiation of highly proliferative cardiac progenitors into primitive CMs that form the heart tube. At ~3 wpc, mesodermal progenitors from the first heart field generate the initial heart tube, and between 3 and 6 wpc, *ISL1*^+^ second heart field progenitors contribute additional myocardium to the arterial and venous poles, driving elongation and looping of the heart tube [96,97]. As progenitors begin to express contractile machinery, their proliferative capacity declines. Consequently, cardiac growth during this stage occurs largely through continued recruitment of newly differentiated CMs from progenitor pools [98,99,100,101].

The second mode of CM growth occurs by cell cycle reentry of primitive CMs. During cardiac looping, CM proliferation becomes regionally heterogeneous. CMs in the outer curvature preferentially re-enter the cell cycle, whereas CMs located at the outflow tract are considerably less proliferative. Trabeculae form as CMs proliferate at the base of the emerging trabeculae. The net result is that the heart balloons into its basic four-chambered architecture with a mesh-like trabecular network that is reminiscent of the amphibian heart. Ventricular compaction begins at ~6 wpc, involving the absorption of trabeculae into the ventricular wall alongside the proliferation of CMs within the compact myocardium. Although CM proliferation continues throughout the heart, recent spatial profiling has shown that cycling CMs are comparatively enriched in the outer ventricular wall at 13 wpc, suggestive of an increasing role for epicardial-derived cues to drive CM proliferation as heart development progresses [102]. While comprehensive time-resolved maps of human CM cycling across development are lacking, comparative studies in mammalian models suggest that CM proliferative activity peaks during the first trimester and declines thereafter (Figure 2A).

### 3.2. Postnatal Cardiomyocyte Proliferation

After birth, human CMs almost entirely exit the cell cycle, yet the heart continues to grow, with ventricular wall thickness linearly increasing 2–3 fold over the first two decades of life [107]. Postnatal CM growth is almost entirely driven by hypertrophy with a seemingly minimal contribution from CM turnover. Several groups have attempted to quantify this low rate of postnatal human CM turnover using marker-based assessments of cell-cycle activity or pulse-chase experiments based on heavy-isotope incorporation (Figure 2B) [104,105,106].

The largest histological analysis of human CM proliferation to date was conducted by Mollova et al. using 40 hearts from donors aged 0 to 59 years [106]. Hearts were obtained from unused transplant donors and cadaveric sources and subjected to strict quality control. CM cell cycle stages were assessed by colocalizing sarcomeric markers (α-actinin and troponin I) with markers of M-phase (H3P) and cytokinesis (MKLP-1) using laser scanning cytometry. The percentage of M-phase CMs was estimated to decline from 0.04% in the first year of life to 0.001% after 40 years of age, while CM cytokinesis was not detected beyond 20 years of age. Integrating these figures with myocardial mass, the number of CMs was estimated to linearly increase by 3.4-fold over the first two decades of life, with turnover rates decreasing from ~4.5% in the first decade of life to ~0.04% after 40 years of age (Figure 2B). Importantly, with a higher rate of cycling compared to cytokinesis across the lifespan, the percentage of polyploid CMs steadily increased from just under 50% during the first year of life to ~60% during the third decade, with minimal change thereafter [106].

An alternative approach to assessing CM turnover is the use of “pulse-chase” types of experiments involving incorporation of heavy elemental isotopes by emerging CMs (“pulse”) followed by retrospective assessment of how many CMs were labeled (“chase”). In their initial study, Bergmann et al. analyzed CM nuclei from 12 cadaveric donors ranging from 19 to 73 years of age for their average ^14^C content using accelerator mass spectrometry [105]. Because cycling CMs incorporate ^14^C in proportion to environmental levels, the rapid decay of atmospheric ^14^C following aboveground nuclear testing in the 1950s and 1960s was used to infer average CM birth dates. After accounting for polyploidy, CM turnover was estimated to decline linearly from ~2% per year at age 20 to ~0.2% per year by age 70 (Figure 2B). Remarkably, despite low rates of annual turnover, cumulative CM renewal was estimated to replace ~45% of CMs postnatally. In a second, larger cohort of 51 donors aged 8–75 years, these estimates were largely confirmed [104]. With more samples from subjects less than 20 years of age, the postnatal CM turnover rate was found to be rapid in early life, decreasing to 0.8% annually by age 20 and further diminishing to 0.3% annually by age 75 (Figure 2B). By age 10, 36% of CMs were estimated to arise postnatally, with only an additional 3% generated by age 75. Similar to Mollova et al.’s results, human CMs were estimated to undergo extensive polyploidization during the second decade of life, coinciding with an increase in CM volume. Overall, ~60% of human CMs are estimated to be polyploid after age 20 [106].

While foundational, prevailing estimates of human CM turnover have several limitations. First, all estimates to date are derived from relatively small cohorts, reflecting the technical constraints associated with acquiring and analyzing high-quality human cardiac tissue. Second, the propensity of postnatal CMs to undergo endomitosis means that only a subset of CMs that enter the cell cycle will result in new CMs. As a result, marker-based approaches could overestimate proliferation rates (see Section 2.2.4). Third, the models used to determine CM turnover are fundamentally dependent on several assumptions. Considering the large size and structural heterogeneity of the human heart, extrapolation of measurements estimated from limited tissue sections compounds any uncertainty. For instance, critical assumptions for the ^14^C pulse-chase approach include the rate of polyploidization over time, the smoothing of ^14^C levels over time, and the time course of atmospheric ^14^C incorporation by new CMs. Work to recapitulate Bergman et al.’s estimates of human CM turnover found that adjusting the rate of polyploidization using a different dataset altered turnover estimates by ~25% and that accounting for up to a 2-year delay in ^14^C incorporation could impact turnover estimates by nearly 4-fold [108]. Taken together, there is clear evidence for a low level of postnatal human CM turnover in the human heart, but the exact abundance and rate remain controversial. While newer strategies such as temporally administered ^15^N to label CMs hold promise, such methods are currently technically and financially impractical to be carried out at a scale to provide true estimates of CM turnover in the population [109,110].

## 4. Therapeutic Heart Regeneration

### 4.1. Cardiac Recovery in Neonatal Humans

Over the years, anecdotal evidence supports a level of cardiac resilience in the neonatal human heart that is not present in the adult human heart. For example, children with an anomalous left main coronary artery that arises from the pulmonary artery have myocardial ischemia with heart failure but generally recover function to normal levels after revascularization [111,112,113]. Similarly, a child with an acute myocardial infarction due to a coronary thrombus with evidence of significant myocardial injury was reported to fully recover function [114]. While provocative, no assessment of CM proliferation has been possible in these scenarios to truly differentiate a regenerative response from recovery of function, from restoration of blood flow to hibernating myocardium, cardioprotection, or another mechanism.

Based on the wealth of animal data showing a capacity for neonatal mammalian heart regeneration and case reports, recent efforts have sought to test the hypothesis that natural CM proliferation can be augmented in the pediatric heart for therapeutic purposes. Tetralogy of Fallot (ToF) is a form of cyanotic congenital heart disease that leads to eventual right ventricular failure. In pre-clinical work, patients with ToF were found to have a defect of CM cytokinesis, associated with hyperactive β-adrenergic signaling [109]. Accordingly, β-adrenergic blockade with propranolol rescued these proliferation defects in cultured CMs. In an ongoing proof-of-concept clinical trial (NCT04713657), 40 infants with ToF will be randomized to receive 1 mg/kg of propranolol four times daily from 1 month of age until reparative surgery, which typically occurs between 2 and 9 months of age [110]. The study is powered to detect a 9% decrease in CM multinucleation with 90% power. CM proliferation will be assessed by administering ^15^N to infants one month (once a day for five days) after the study treatment to pulse cycling CMs and to then use Multi-isotope Imaging Mass Spectrometry (MIMS) on right ventricular samples collected at the time of surgery. Other endpoints include assessments of right ventricular size and mass. The trial is expected to conclude by December 2030.

### 4.2. Cardiac Recovery with Mechanical Unloading

Left ventricular assist devices (LVADs) are surgically implanted pumps that draw and propel blood from the left ventricle to the systemic circulation. LVADs are used in patients with end-stage heart failure, and in <5% of cases, recovery of native ventricular function occurs such that the LVAD can be explanted [115]. Several clinical trials have demonstrated that with a deliberate intention to recover strategy consisting of intense administration of heart failure therapeutics, rates of durable recovery can occur in up to 40% of carefully selected LVAD recipients [116]. Even in cases where full recovery does not occur, partial recovery can still improve outcomes, with reduced bleeding risk and a lower need for hospitalization [117]. The mechanisms for cardiac recovery remain under investigation, but several reports have suggested that CM proliferation may occur. In a histological assessment of patients supported by an LVAD who later went on to heart transplant, CM cycling was reported to increase preferentially in individuals with a non-ischemic cardiomyopathy [118]. More recent work using PET scanning and ^14^C labeling suggests an increase in cardiomyogenesis in patients who recover in response to LVAD support [86,119,120]. Using the “bomb-pulse” ^14^C method, the rate of CM turnover was estimated to increase to an astounding 3.1% per year with mechanical unloading [119]. However, the same methodological assumptions underlying the original estimates of CM turnover by Bergman et al. are applicable to this dataset as well (see Section 3.2). Thus, while provocative, whether bona fide cardiomyogenesis occurs after LVAD support is an open question. As with recovery of ventricular function itself, there is some heterogeneity amongst centers in the ability to identify CM cycling with LVAD support and whether PET evidence of cardiomyogenesis occurs [86,121]. Moreover, whether the low level of CM proliferation that has been suggested to occur with LVAD support can account for the functional recovery of LVAD “responders” is unclear, as reverse remodeling, changes in myocardial energetics, changes in CM handling of calcium, regression of pathologic hypertrophy, and other non-regenerative mechanisms could also explain functional recovery [122,123,124]. Nevertheless, the potential for LVAD support to enable a permissive environment for CM proliferation is an interesting concept that needs to be further explored.

### 4.3. Emerging Regenerative Therapeutics

#### 4.3.1. MicroRNAs

**Figure 3:**
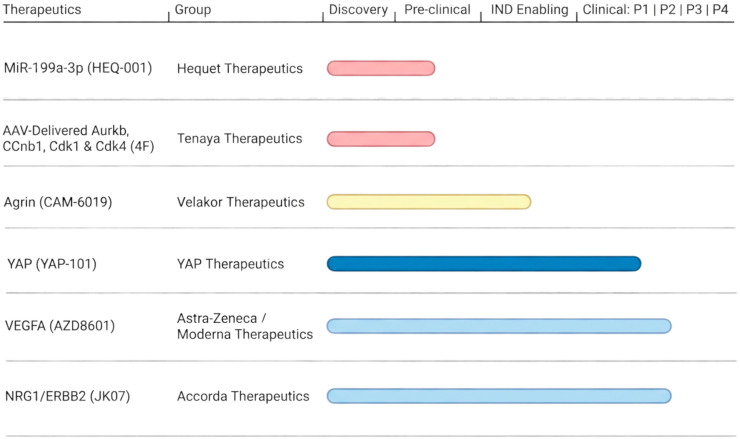
The pipeline for therapeutics to augment human CM proliferation. Bars indicate the latest clinical stage for the indicated intervention. Created in BioRender. McLane, R. (2026) (https://BioRender.com/ywvh2xr) licensed under CC BY 4.0) (accessed on 28 January 2026).

After performing high-content screening for effects on proliferation of 875 miRNA mimics in neonatal rat CMs, hsa-miR-590-3p and hsa-miR-199a-3p were identified as being able to stimulate cycling in adult CMs in vitro and in vivo [125]. To enable efficient myocardial gene delivery to mice in vivo, miRs were delivered via intramyocardial injection of adeno-associated virus (AAV) serotype 9, which efficiently targets myocardium [126]. While overexpressing these miRs at the time of coronary ligation resulted in a modest amount of CM cycling (~0.9% by EdU incorporation), cardiac function nearly normalized, and infarct size was reduced by approximately 50%, suggestive of a simultaneous regeneration-independent benefit. Subsequent experiments showed that a single injection of either miR as a synthetic construct complexed with an RNA transfection agent could similarly boost CM cycling and improve cardiac function after coronary ligation, with hsa-miR-590-3p being more potent in rat cells and hsa-miR-199a-3p being more potent in mouse cells [127]. Mechanistically, these miRs appear to exert their effects through the YAP pathway [128]. Interestingly, when AAV encoding hsa-miR-199a-3p was injected into the left ventricular wall after experimental MI in pigs, cardiac function and scar size progressively improved along with increased CM cycling compared to control AAV. However, at 7–8 weeks post-treatment, 70% of miR-199a-3p-treated pigs died of sudden death with evidence for ventricular fibrillation compared to none in the control group [37]. miR-199a-3p is currently under development as HEQ-001 by Heqet Therapeutics (Torino, Italy) but remains in pre-clinical development likely navigating arrhythmic concerns (Figure 3) [129].

#### 4.3.2. Transient Cell Cycle Activators

To determine which cell cycle genes best induce CM proliferation, Mohamed et al. screened an adenoviral library of 15 cell cycle regulators dynamically expressed in the embryonic, neonatal, and adult mouse heart [91]. Of the factors tested, *Aurkb*, *Ccnb1*, and *Cdk1* were able to stimulate CM cycling of post-mitotic mouse CMs and human iPSC-derived CMs that had been cultured for an extended period of time. The addition of *Cdk4* to this cocktail (4F) limited the death of cycling CMs. Together, when 4F was injected into mice at the time of coronary ligation, cardiac function improved, with rigorous evidence for bona fide CM proliferation by use of the Mosaic Analysis of Double Markers (MADM) lineage tracing system {Mohamed, T.M.A. et al., 2018}. Analogous studies in rats with follow-up through 16 weeks also demonstrated a functional benefit, without evidence of obvious ventricular arrhythmias on short-term ECG screening or gross evidence of systemic tumorigenesis [130]. Porcine studies showed similar results and notably no sudden death, but functional benefits in pigs treated with 4F were less than those reported with the miR-199a-3p and follow-up was for 4 weeks, less than the 7–8 weeks when sudden death was observed with miR-199a-3p [130]. AAV delivery of 4F is suggested to be under development as a regeneration strategy by Tenaya Therapeutics (San Francisco, CA, USA) and remains at the preclinical stage (Figure 3) [131].

#### 4.3.3. Agrin

Agrin is an extracellular matrix protein known to assist in the formation of the neuromuscular junction. In an in vitro screen for extracellular matrix proteins that could stimulate CM proliferation, Agrin was identified as a protein that is developmentally down-regulated in the postnatal heart [132]. Genetic deletion of *Agrin* was reported to impair heart regeneration of neonatal mice. In adult mice following left anterior descending artery (LAD) ligation, a single intramyocardial dose of recombinant Agrin reduced the scar area beginning around day 14, with a significant reduction of nearly 30% by day 35. These changes were accompanied by a ~10% absolute increase in left ventricular ejection fraction (LVEF) versus placebo at late follow-up [132]. Like with hsa-miR-590-3p and hsa-miR-199a-3p, the functional benefits associated with Agrin treatment are likely to go beyond an increase in Ki67^+^ CMs from 0.2 to 0.8%, also involving non-regenerative mechanisms [132]. Similar effects were also reported in a porcine ischemia/reperfusion model after treatment with Agrin [36]. Here, no arrhythmic events were reported, but pigs were also only followed for 4 weeks. Towards efficacy in humans, culture of EHTs made from hiPSC-CMs had increased CM proliferation after 2 weeks of recombinant Agrin [132]. Agrin is thought to exert its salubrious effects through dissociation of the dystrophin-glycan complex to enable translocation of Yap to the nucleus in CMs and by promoting fibroblast senescence [132,133]. Recombinant Agrin is currently being developed as CAM-6019 by Velakor Therapeutics and is in pre-clinical testing after completing IND-enabling studies (Figure 3) [134].

#### 4.3.4. VEGFA

In zebrafish and neonatal mice, rapid revascularization of injured myocardium is required for tissue regeneration [135,136,137]. Augmenting this revascularization response with VEGFA boosts CM proliferation in zebrafish and neonatal mice [15,26]. Although a number of clinical trials have failed to demonstrate any cardiac benefits by VEGFA overexpression, genetic modeling predicts that VEGFA can enhance human cardiac growth similarly to zebrafish and neonatal mice [26]. Moreover, recovery of ventricular function in patients with heart failure is associated with higher circulating levels of VEGFA [138].

Because early attempts to use VEGFA therapeutically relied on recombinant protein or injection of plasmid DNA encoding *VEGFA* into the heart, these studies may have been limited by little to no durable expression of *VEGFA* [139,140]. Newer generation therapeutics have focused on improved delivery methods, such as modified mRNA which substitutes uridine for pseudouridine to reduce immunogenicity and to improve stability [141]. In pre-clinical murine MI models, intramyocardial injection of VEGFA-modRNA at the time of coronary artery ligation increased capillary density and reduced apoptosis and scar burden, resulting in improved left ventricular function compared to a modRNA encoding luciferase control [141]. In a porcine permanent occlusion MI model, intracardiac injection of VEGFA-modRNA at 7 days post-MI modestly improved LVEF by ~4–5% at 2 months and reduced the mid-ventricular infarct area by ~5–6% compared to vehicle [142].

VEGFA-modRNA is now being developed as AZD8601 by AstraZeneca and Moderna Therapeutics. In the phase 2a EPICCURE study (NCT03370887), 11 patients with an ischemic cardiomyopathy undergoing coronary artery bypass grafting (CABG) received 30 injections of VEGFA-modRNA (3 mg total) or placebo into viable myocardium mapped by quantitative PET at the time of surgery (Figure 3) [143,144]. At 6 months, no treatment-related serious adverse events were identified. As in porcine studies, VEGFA-modRNA treatment resulted in about 4% increase in LVEF versus the placebo group. Stress myocardial blood flow increased after CABG in both arms, but there was no significant between-group difference in myocardial blood flow or perfusion reserve at follow-up, suggesting room for dosage optimization. Overall, while VEGFA-modRNA was generally associated with positive outcomes, the small sample size of EPICCURE limits any definitive conclusions. No attempts to assay heart regeneration have been assessed as a part of either pre-clinical or clinical work with VEGFA-modRNA. As of this writing, no future studies have been announced for AZD8601.

#### 4.3.5. NRG1/ERBB Signaling

NRG1 is a member of the EGF family of growth factors that works through ErbB2 and ErbB4 receptors on CMs to stimulate CM proliferation during heart development. Genetic deletion of *Nrg1*, *Erbb2*, or *Erbb4* in mice all lead to embryonic lethality with thinned, hypoplastic hearts [145,146]. A possible role for NRG1 in regeneration was reported in 2009, when NRG1 was reported to increase CM proliferation in vitro and then promote recovery of ventricular function after experimental MI in adult mice [147]. Since then, the window for NRG1 effectiveness has been determined to be more nuanced, with the ability to induce cycling possibly related to a developmental window when *Erbb2* is expressed in CMs [31,148]. Along with this idea, a constitutively activated Erbb2 receptor drives the proliferation of CMs in adult mice, with the caveat that prolonged expression leads to cardiac dysfunction and CM immaturity. Whether such developmental gating of ERBB2 occurs in the human heart is unclear, as *ERBB2* expression by adult CMs in humans is thought to be the basis for the cardiotoxicity observed with the anti-Erbb2 drug trastuzumab in some patients with breast cancer. Of note, NRG1 likely has multiple effects on the heart, such as cytoprotection by activating PI3K/AKT survival pathways, improving mitochondrial membrane potential, and mitigating stress-induced apoptosis [149,150].

The activation of NRG1/ERBB2 signaling has been studied clinically in two different clinical programs. A first-in-human, double-blind, placebo-controlled single ascending dose phase I trial (NCT01258387) using a recombinant NRG1 formulation called cimaglermin alpha (Accorda Therapeutics) was conducted in 40 patients with left ventricular dysfunction (LVEF < 40%) [151]. A relative improvement in LVEF was noted 90 days after the infusion, with a magnitude that approximated a dose–response relationship. Based on these data, a phase 1B study was planned. However, a dose-limiting toxicity event with an increase in liver enzymes at the highest dose in one patient resulted in a clinical hold on the program, which was ultimately lifted. However, additional trials were never resumed.

A second therapeutic strategy has been to focus on selectively driving Erbb2/Errb4 signaling with an engineered biologic, JK07 (SaubrisBio, Gaithersburg, MD, USA), that fuses recombinant Nrg1 with an Erbb3 blocking antibody. A single ascending dose study in 44 patients with NYHA class II-III heart failure showed a significant increase in LVEF at the two highest doses tested, accompanied by reverse cardiac remodeling (NCT04210375) [152]. Treatment-related adverse events were seen with higher doses, but dose-limiting toxicity was not observed. Interestingly, participants who received JK07 were reported to have an acute increase in LVEF within days, suggesting a CM proliferation-independent mechanism. JK07 is currently being evaluated in the phase 2 RENEU-HF trial (NCT06369298) in patients with heart failure with reduced ejection fraction and in patients with heart failure with preserved ejection fraction and underlying atrial fibrillation. The results are expected in 2026 (Figure 3). Common to both studies, aside from a possible increase in cardiac function, was a rise in NT-proBNP, possibly paralleling the loss of CM maturity observed in animal models.

#### 4.3.6. Hippo/YAP Signaling

The Hippo pathway is a genetically conserved system that regulates organ size during development by inhibiting activation of the transcription factor YAP to constrain growth. Deletion of the Hippo kinase *Salvador* from CMs during development in mice leads to cardiomegaly due to excessive CM proliferation, while deletion of YAP leads to hypoplastic hearts with diminished CM proliferation [153,154,155]. Subsequently, efforts to deactivate Hippo signaling or increase YAP transcriptional activity have emerged as leading strategies for therapeutic heart regeneration. Indeed, both strategies are able to induce CMs to proliferate and regenerate after experimental myocardial infarction in adult mice [156,157,158]. Remarkably, using an AAV encoding a shRNA for *Salvador* knockdown three weeks after coronary ligation in mice or two weeks after coronary ligation in pigs is able to restore cardiac function with evidence for CM proliferation, suggesting that YAP activation is beneficial even with long-standing chronic cardiomyopathy [35,156]. However, prolonged YAP-mediated proliferation is deleterious. In mice engineered to express a constitutively active YAP-5SA, sudden death occurs within one week of transgene induction [159,160].

Because the Hippo pathway is activated in human heart failure samples, YAP activation is a particularly promising approach for therapeutic heart regeneration [156]. Medley Therapeutics (Laguna Hills, CA, USA) is developing an AAV-based treatment to deliver an shRNA against *Salvador* under the control of a CM-specific promoter as YAP101. In their ongoing phase I, open-label Salvador-HF study (NCT06831825), 24 patients with an ischemic cardiomyopathy will receive one of four ascending doses of YAP101 via endocardial injection into the myocardium (Figure 3) [161]. This dose-finding study is meant to determine the maximally tolerated viral dose and to assess for dose-limiting toxicities. Secondary endpoints include changes in ventricular function along with standard heart failure outcomes such as the need for hospitalization, transplantation, or LVAD. Recently, this study passed its first milestone, with no dose-limiting events in the first cohort of three patients dosed with YAP101 [162].

## 5. Challenges for Therapeutic Heart Regeneration

### 5.1. Arrhythmic Risk

Work over the past decade has clearly established that uncontrolled CM proliferation can be toxic. Expression of a constitutively active *ErbB2* leads to death in neonatal, juvenile, and adult mice, with evidence for thickened hearts and a compromised stroke volume, similar to pathologic hypertrophic cardiomyopathy [31]. Meanwhile, transgenic expression of a constitutively activated YAP leads to death within a week of transgene activation, suggestive of an arrhythmogenic phenotype [159,160]. Similarly, pigs dosed with *miR199a* experience sudden cardiac death, with evidence for ventricular fibrillation [37].

Risk of arrhythmia may be fundamentally linked to CM proliferation. In zebrafish, regenerating hearts have altered calcium handling [8,163]. Similarly, in iPSC-CMs, membrane voltage changes with sarcomeric disassembly, a required event for CM proliferation [130]. Thus, regenerative therapeutics will likely need to mitigate arrhythmias, as with transplantation of iPSC-CMs, where adjunctive antiarrhythmic therapy with amiodarone and ivabradine has shown promise in reducing arrhythmia burden [164].

One option to reduce the risk of CM proliferation is to use approaches that only transiently promote CM proliferation. For instance, transient expression of a constitutively active *Erbb2* restores function after experimental MI [31]. Similarly, AAV encoding a shRNA against Salvador is thought to be transient and is well tolerated after MI [35]. Several approaches are being developed to ensure transient kinetics. The first is to use short-acting molecules such as modified RNAs [141,165,166,167]. The second has been to take advantage of the natural regenerative machinery in zebrafish, which halts CM proliferation once organ function is restored. Tissue Regeneration Enhancer Elements (TREEs) have been identified that are activated with injury and deactivated after regeneration [20,168]. TREEs have been used to drive pro-proliferative transgenes like a constitutively activated YAP in mice without apparent toxicity [169]. Finally, more recently, an AAV system that activates payload expression in response to the small molecule LMI070 has been used to transiently express a constitutively activated YAP [170]. While promising, even transient proliferation will need to be assessed for rhythm disturbances, especially when considering that the target population of regenerative therapeutics has a heightened arrhythmic risk.

### 5.2. Gene Delivery

Nearly all therapeutic strategies being attempted for therapeutic heart regeneration require direct injection into the myocardium. While direct access occurs at the time of cardiac surgery, the most common approach for accessing myocardium is by way of a percutaneous injection catheter. Such catheters were originally developed for cell therapy applications but have been repurposed for cardiac gene therapy and are well tolerated, with few adverse events reported. A second approach with the potential to be non-invasive is the use of AAV. Conventional serotypes have low tropism for the heart, but in recent years several capsids have been evolved for better cardiac transduction [171,172,173,174]. However, AAV has significant limitations around immunogenicity, packaging capacity, and off-target effects, especially in the liver [175]. Lipid nanoparticles are an alternative, but all commercially available LNPs primarily target the liver when given intravenously. Recently, a new strategy to detarget the liver by interfering with the ApoE corona around LNPs seems to promote cardiac gene delivery [176]. Finally, synthetic RNAs have been developed using a bipartite “suppress the suppressor” approach for CM specific translation. The first RNA encodes L7AE but contains a sensor for the CM-specific *miR208*, such that when *miR208* is present, L7AE translation is suppressed. The second RNA contains the gene of interest after a K motif. When L7AE is present, it binds the K motif and prevents translation. However, in CMs where L7AE translation is suppressed by *miR208*, translation of the gene of interest is no longer inhibited, resulting in CM-specific delivery of the payload [165,166].

### 5.3. Oncogenic Risk

Because most interventions at the forefront of therapeutic heart regeneration are mitogens, they have the potential to activate proliferation of cell types beyond CMs. Indeed, Nrg1/Errb2, VEGFA, and YAP signaling have all been linked to various malignancies, and their inhibition is the basis for many cancer therapies. Thus, one potential toxicity of regenerative therapeutics is off-target malignancy. While strategies such as transient factor expression, targeted delivery of factors to the heart, and CM-specific factor expression can reduce this risk, long-term follow-up of patients for malignancy will likely be needed.

### 5.4. Trial Design

As the prospect of translating cardiac regeneration biology to therapeutics gets closer, trial design needs to be considered. The first consideration is that of the patient population. While patients with long-standing dilated cardiomyopathies with high scar burden would be the ultimate target population, children with heart failure or LVAD recipients may harbor CMs that are more permissive to regenerative strategies, potentially allowing for early success [110,119]. The second consideration is that of safety. In light of safety signals for sudden cardiac death, target populations will need close monitoring for arrhythmogenicity and may require internal cardiac defibrillators or adjunctive antiarrhythmic treatments to be considered as part of study protocols. A third consideration is one of trial endpoints. Clinical endpoints for patients with heart failure typically focus on mortality, rates of hospitalization for heart failure, and quality of life measures. To be adopted into clinical practice, regenerative therapeutics will need to improve these outcomes compared to the standard of care. Such studies typically involve hundreds of patients. Secondary clinical endpoints often look at biomarkers, such as NT-proBNP. However, with the extent of CM dedifferentiation that is expected to occur with CM proliferation, NT-proBNP and high-sensitivity troponin levels might increase independently of progressive heart failure. Finally, how to truly assess human heart regeneration will be a challenge. Strictly speaking, tissue regeneration is the replacement of lost or dysfunctional tissue with new, functional tissue. In pre-clinical systems, regeneration is often assessed by assaying tissue specimens for markers of cycling, DNA content, or clonal analysis. Strategies such as ^15^N pulse, as being used in the ToF trial, are not feasible without adequate access to myocardial tissue [110]. For the majority of patients, regeneration will likely need to be assumed by recovery of left ventricular function and a decrease in scarring, indicating that serial imaging with cardiac MRI or PET scanning may be required. Interestingly, about 20% of patients with heart failure recover function in response to current medical treatment [4]. However, such recovery is not a cure, as cessation of medical therapy is associated with a high relapse rate of disease [4]. Thus, one potential consideration for true regenerative therapeutics would be the ability to successfully wean patients off heart failure therapies.

## 6. Future Directions in Cardiac Regeneration

The successful translation of regenerative biology to patients hinges on bridging the gap between successful proof-of-concept studies in model systems and achieving consistent, durable benefits in human disease. Progress in cardiac regeneration will depend not on a single breakthrough but on integrating multidisciplinary innovations, from the discovery of new regenerative therapeutics, to the development of better cardiac delivery strategies, to the development of new methods sensitive enough to detect and quantify regenerative responses. While astounding progress in heart regeneration has been made, translational challenges remain. As such, future work presents an opportunity to transition regenerative cardiology from experimental promise toward carefully validated clinical application over the next decade

## Data Availability

Not applicable.

## Abbreviations

AAV	Adeno-associated virus
CABG	Coronary artery bypass grafting
CM	Cardiomyocyte
ECM	Extracellular matrix
EHTs	Engineered heart tissues
iCMs	Human induced pluripotent stem cell-derived cardiomyocytes
iPSC	Induced pluripotent stem cell
LVAD	Left ventricular assist device
LVEF	Left ventricular ejection fraction
MIMS	Multi-isotope imaging mass spectrometry
miRNA	MicroRNA
modRNA	Modified messenger RNA
PSC	Pluripotent stem cell
ToF	Tetralogy of Fallot
wpc	Weeks post-conception
